# What are These Cysts Doing in My Graft? A Meta-Analysis on Cystic Occurrence After Autografting and Allografting for Osteochondral Lesions of the Talus

**DOI:** 10.1177/19476035251333374

**Published:** 2025-04-15

**Authors:** Jari Dahmen, Julian J. Hollander, James J. Butler, Kaj S. Emanuel, Quinten G.H. Rikken, Sjoerd A.S. Stufkens, John G. Kennedy, Gino M.M.J. Kerkhoffs

**Affiliations:** 1 Department of Orthopedic Surgery, Amsterdam Movement Sciences, Academic Medical Center, Amsterdam UMC, Location AMC, University of Amsterdam, Amsterdam, The Netherlands; 2 Academic Center for Evidence Based Sports Medicine, Amsterdam UMC, University of Amsterdam, Amsterdam, The Netherlands; 3 Amsterdam Collaboration for Health and Safety in Sports, International Olympic Committee Research Center, Amsterdam UMC, University of Amsterdam, Amsterdam, The Netherlands; 4 Department of Orthopedic Surgery, NYU Langone Health, New York, NY, USA

**Keywords:** talus, ankle, joint involved, osteochondral, basic science articular cartilage, basic science, clinical research ankle, clinical research

## Abstract

**Background:**

The exact incidence of cyst formation after graft transplantation for osteochondral lesions is unknown. The primary purpose was to assess and compare cystic occurrence after autografting, allografting, and osteoperiosteal grafting for osteochondral lesions of the talus. Our secondary aim was to assess the correlation of clinical outcomes with the presence of postoperative cysts.

**Methods:**

A literature search was performed up to October 2023 through PubMed, Embase (Ovid), and Cochrane Library. The primary outcome was the postoperative cystic occurrence rate. A random-effects model with moderator analysis was used to calculate differences in occurrence rates between treatment groups. The relationship between the presence of cysts and clinical outcomes was described.

**Results:**

Thirteen studies were included with 382 ankles. The average radiological follow-up at which the presence of cystic occurrence was assessed ranged from 12 to 84 months. The rates of cystic occurrence for the osteochondral autograft transplantation group, the allograft transplantation group, and the osteoperiosteal transplantation group were 42% (95% confidence interval [CI] = 24-61), 58% (95% CI = 40-74), and 34% (95% CI = 12-67), respectively, without any significant differences noted. No relationship between the presence of cysts and clinical outcomes was found.

**Conclusion:**

Postoperative cystic occurrence is common after osteochondral autograft transplantation (42%), allograft transplantation (58%), and osteoperiosteal transplantation (34%) in osteochondral lesions of the talus—without significant intertreatment differences. The postoperative presence of cysts was not correlated with clinical outcomes. Future research should assess whether the postoperative presence of cysts correlates with (clinical) outcomes at longer follow-up.

**Level of Evidence:**

Level IV, systematic review and meta-analysis.

## Introduction

An osteochondral lesion of the talus (OLT) is defined as damage to the talar cartilage and its underlying subchondral bone,^1^ often caused by ankle trauma.^2,3^ These lesions typically manifest with nonspecific symptoms thus are often not identified due to a low index of suspicion, leading to debilitating pain and impaired joint function. Surgical interventions have been shown to be clinically effective treatment options,^4,5^ with autograft procedures, allograft procedures, and osteoperiosteal autograft procedures typically recommended for larger lesions.^6,7^ Grafting procedures ensure high-quality bone and/or cartilage restoration, and satisfactory clinical outcomes have been reported at mid-term follow-up.^4,5,8-12^ However, the development of cysts within the transplanted graft has been frequently seen as a complication or adverse event after the surgery.^9,10,13,14^ It is unclear whether certain transplantation techniques produce a significantly higher or lower rate of cystic development at short-term, mid-term, and long-term follow-up, nor is it certain if the presence of postoperative cyst affects clinical outcomes. Several factors may play a role in the development of these cysts including but not limited to subchondral bone alterations, immune reactions (in case of allografts), quality of graft integration, postoperative rehabilitation, and/or potential (over)loading of the joint specifically where the graft is implanted.^15-18^

The primary research aim of the present meta-analysis is, therefore, to provide a comprehensive assessment of the rate of development of postoperative cysts within transplanted grafts following treatment of OLTs. We aim to quantify the prevalence of cyst formation after autograft procedures and allograft procedures for the treatment of OLTs and investigate potential variations in cyst development rates between these surgical techniques. The secondary research aim is to assess the potential correlation between the presence of cysts and patient-reported outcome measures (PROMs) to determine whether cyst formation is linked to inferior clinical outcomes.

The findings of the study will be of clinical relevance as the outcomes will form a strong basis toward further exploring the etiology behind postoperative cyst formation, studying the correlation between complaints (pain and/or other PROMs) and the presence of cysts, and guiding physicians to further improve the treatment outcomes for patients with large osteochondral lesions (OCLs) of the ankle.

## Methods

A systematic review of the literature was performed and was reported according to the 2020 methodology of the Preferred Reporting Items for Systematic Reviews and Meta-Analyses (PRISMA).^19^

### Search Strategy

The PubMed, EMBASE (Ovid), and Cochrane Library databases were searched from inception up to October 24, 2023, for potentially eligible articles. The full search strategy is outlined in Appendix A.

### Study Selection and Eligibility Criteria

Two independent reviewers (JD and JH) screened titles and abstracts using predefined criteria in Rayyan.^20^ Subsequently, articles were screened for full text and included when they met the inclusion criteria (**Table 1**). Discordant judgment in study inclusion was resolved by consensus discussion together with a third reviewer (GK).

**Table 1.** Inclusion and Exclusion Criteria.

Inclusion	Exclusion
Clinical studies assessing and describing the presence of occurrence of postoperative cysts after osteochondral autograft transplantation, osteochondral allograft transplantation, and en-bloc osteoperiosteal transplantation for osteochondral lesions of the talus with a mean minimum radiological (MRI/CT only) follow-up of 6 months after the transplantation procedure	Studies that did not present original data (e.g., reviews, technique papers, letters to the editor, editorial)
A cohort of 5 or more patients needed to have had a follow-up MRI/CT during which the presence of cysts was specifically scored	Conference abstracts
Written in English	Animal or cadaveric studies
Full-text available	Double publication including overlap of patients

### Methodological Quality Assessment

Methodological quality of the studies was evaluated using the validated Methodological Index for Non-Randomized Studies (MINORS) criteria^21^ by 2 independent reviewers.

### Data Extraction

The following study characteristics were retrieved: authors, year of publication, and study design. Also, the following patient characteristics were retrieved: sex, age, number of lesions, number of ankles, lesion size and/or area, lesion location, type of index surgical intervention, timing of postoperative radiological (magnetic resonance imaging [MRI]/computed tomography [CT]) assessment, and methods for clinical- and radiological assessment and their corresponding outcomes. The presence of postoperative cyst occurrence (including the description of localization of the cysts, i.e. within the transplant, elsewhere, or not described) was extracted as well as any correlation analyses between the presence of postoperative occurrence of cysts and clinical outcomes. Subanalyses present in the publications on intra-treatment comparative analyses were also extracted (e.g., studying the comparative effect of the intraoperative addition of biologicals).

### Statistical and Data Analysis

It should be noted that in the case of presence of both a postoperative CT scan as well as an MRI-scan assessing the presence of postoperative occurrence of cysts, the CT scan was chosen as the main radiological parameter to base the analyses on.^22-24^ In the case of multiple radiological follow-up moments in time, the latest moment of follow-up was chosen. Furthermore, whenever there were multiple publications with an overlap of patients studied in the publications, the strategy of the authors was to choose the publication(s) yielding the highest number of patients for analysis. Another important methodological aspect to mention is that whenever the authors of the included studies mentioned both a postoperative presence of intra-transplant and outside-transplant cysts as assessed with the follow-up MRI and/or CT scan, we chose to solely extract the intra-graft cyst occurrence rate. All included patients were assigned to treatment groups using the grouping method as previously described by Steman *et al*.^25^

#### Primary outcome measure

The primary outcome was the postoperative occurrence rate of cyst formation. The different occurrence rates of cysts were pooled using a random-effects model. These analyses were performed in an analysis model using R version 4.1.0 (R Core team, Vienna, Austria; metaprop function from the meta package).^26^ The Clopper-Pearson interval was used to assess 95% confidence intervals (CIs). Differences in cystic development rates were compared among the following treatment groups using a moderator test for subgroup analysis with an α < 0.05 indicating statistical significance: osteochondral autograft transplantation, osteochondral allograft transplantation, and osteoperiosteal autograft transplantation.^27^

#### Secondary outcome measures

One of the secondary outcome measures was the explorative description of present relationships between the presence of postoperative cysts and clinical outcomes. Another secondary outcome measure was the explorative description of present relationships between the presence of postoperative cysts and patient or treatment-related factors.

## Results

### Search Results

**Figure 1.**
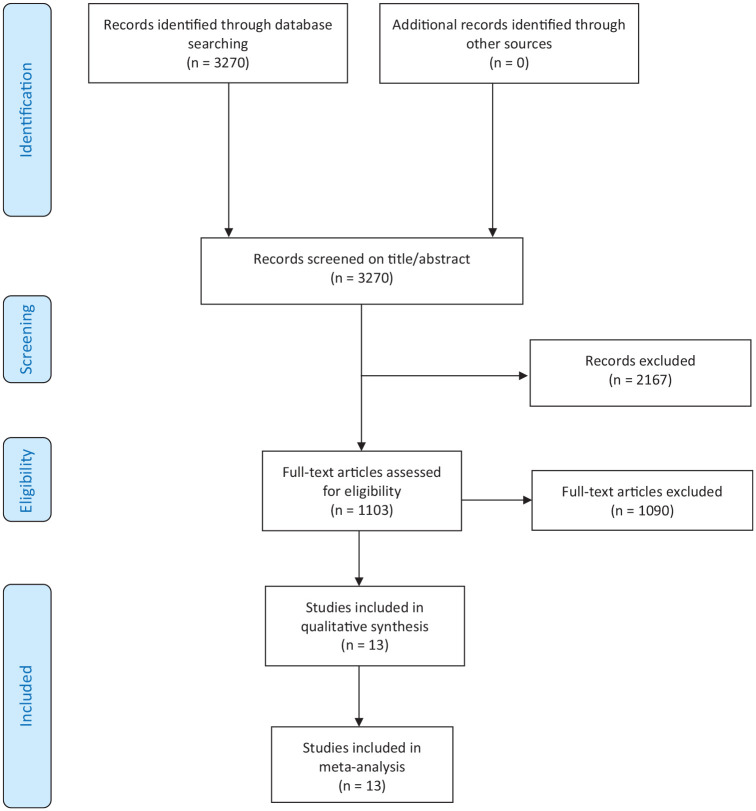
PRISMA flow diagram.

A total of 1,103 articles were included for full-text screening. A total of 13 articles were included for analysis (**Fig. 1**).^13,14,28-38^

### Methodological Quality

The overall methodological quality scores with the MINORS-criteria for comparative and noncomparative studies are shown in Appendix B.

### Study Characteristics

The 13 studies reported a total of 438 ankles, of which 382 patients (88%) underwent postoperative MRI and/or CT after the auto- or allograft transplantation. The patients received the following surgical treatments: osteochondral autograft transplantation (*n* = 247), osteochondral allograft transplantation (*n* = 31), and osteoperiosteal transplantation (*n* = 104). The mean or median radiological follow-up at which the presence of cystic occurrence was assessed ranged from 12 to 84 months postoperatively (**Table 3**). Six^28-31,33,34^ of 13 studies did not specify whether the postoperative cystic occurrence was specifically localized within or outside of the transplant, while 7^13,14,32,35-38^ of the 13 studies specifically stated that the cyst occurrence was within the transplant with one^38^ of these 7 studies reporting on the occurrence rate of both the presence of cysts within the transplant and outside of the transplant. Other study and demographic patient characteristics are included in **Tables 2** and **3**. **Table 3** also described whether the authors reported whether the found cysts were located in or outside of the implanted graft.

**Table 2.** Study Characteristics and Patient Demographic Characteristics Part 1.

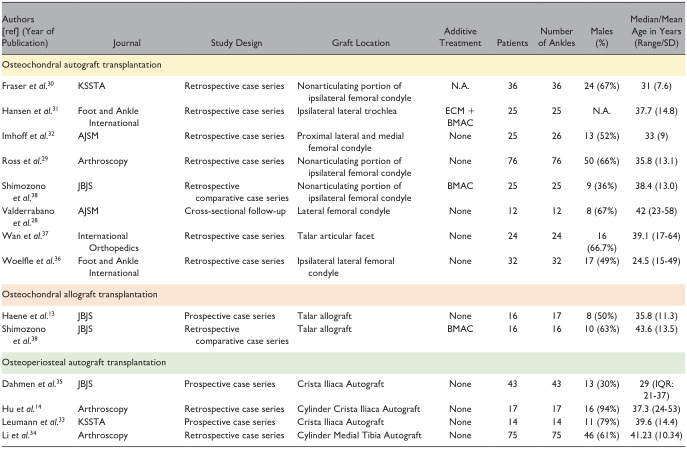

Abbreviations: BMAC, bone marrow aspirate concentrate; CT, computed tomography; ECM, extracellular matrix; MRI, magnetic resonance imaging; N.A., not available; SD, standard deviation.

**Table 3.** Study Characteristics and Patient Demographic Characteristics Part 2.

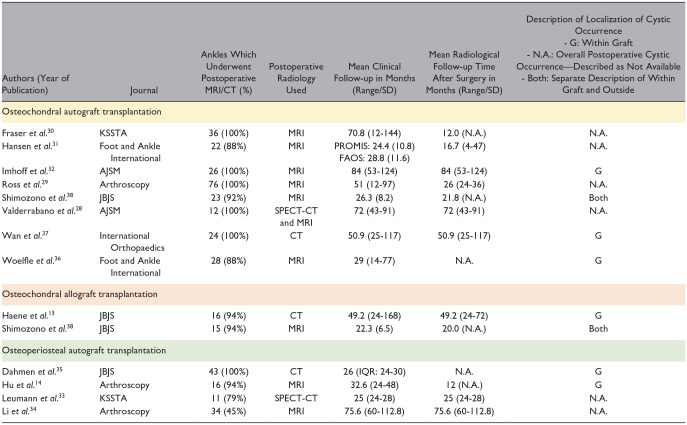

Abbreviation: CT, computed tomography; MRI, magnetic resonance imaging; N.A., not available; SPECT-CT, single photon emission computed tomography–computed tomography; SD, standard deviation.

### Postoperative Cyst Occurrence Rate

**Figure 2.**
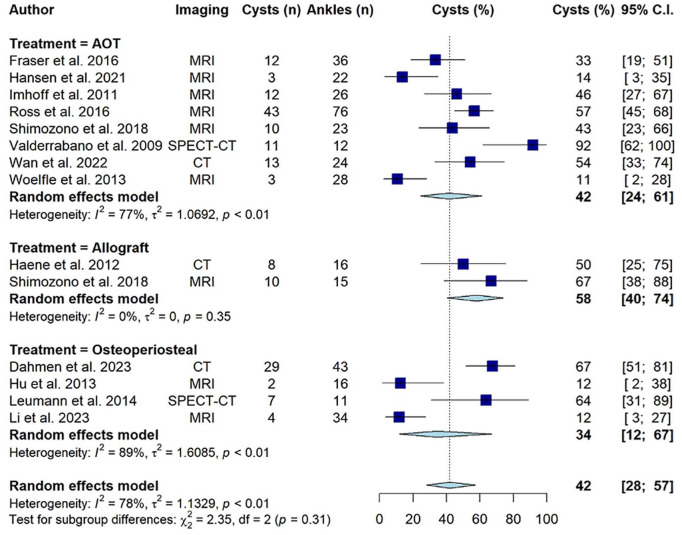
Forest plot of included studies. Abbreviation: AOT, osteochondral autograft transplantation; CI, confidence interval; CT, computed tomography; MRI, magnetic resonance imaging.

The rates of postoperative cyst occurrence for the osteochondral autograft transplantation group, the allograft transplantation group, and the osteoperiosteal transplantation group were 42% (95% CI = 24-61), 58% (95% CI = 40-74), and 34% (95% CI = 12-67), respectively, without any significant differences noted between these treatment groups (*P* = 0.31, **Fig. 2**). The overall rate of postoperative cyst development was 42% (95% CI = 28-57) (**Fig. 2**).

### Relationship Between Postoperative Cysts and Clinical Outcomes

Six studies^28-30,35-37^ described the relationship between the presence of postoperative cysts and clinical outcomes. No studies found a significant relationship between the presence of cysts and patient-reported clinical outcomes (**Table 4**).

**Table 4.** Study Outcomes.

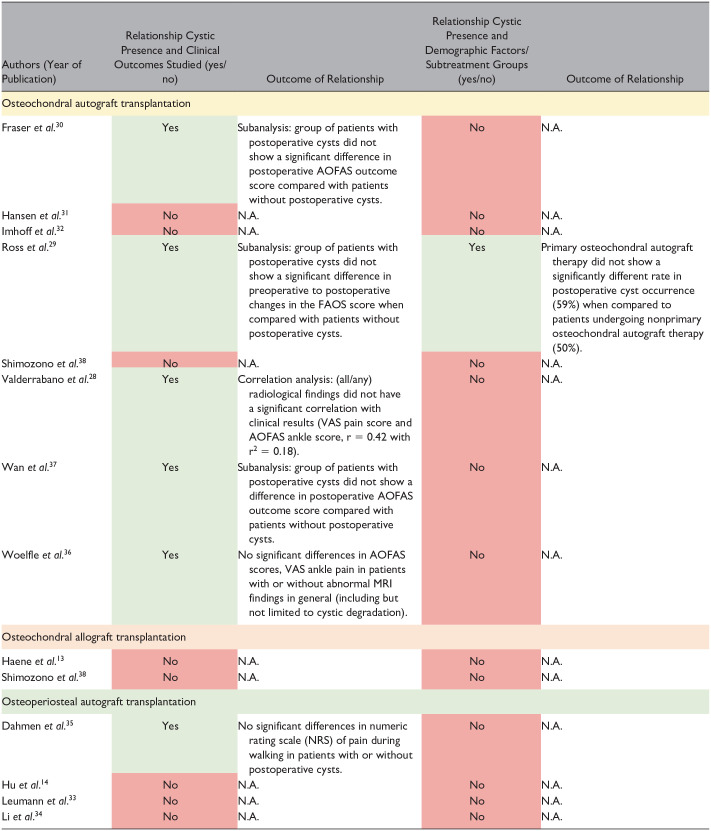

### Relationship Between Postoperative Cysts and Treatment Aspects or Other Nonclinical Outcome-related Factors

One study (Ross *et al*.^29^) of the total 13 studies (8%) evaluated the presence of a potential relationship between cyst presence and other nonclinical outcome-related variables (**Table 4**). Ross *et al*.^29^ assessed whether primary osteochondral autograft therapy showed a different rate in postoperative cyst occurrence when compared to patients undergoing nonprimary osteochondral autograft therapy: no significant differences were found.

## Discussion

The most important finding of this current systematic review and meta-analysis is that postoperative cyst formation is a common finding following osteochondral autograft transplantation (42%), allograft transplantation (58%), and osteoperiosteal graft transplantation (34%) for the management of OLTs. There did not appear to be a statistically significant difference in the prevalence of these cysts between different surgical treatment techniques (*P* = 0.31). More so, the postoperative presence of cysts was not correlated with inferior clinical outcomes in any of the included studies. These findings provide valuable insights into the prevalence of postoperative cysts and shed light on the clinical relevance of cyst development in patients undergoing osteochondral/periosteal autograft and allograft procedures for OLT.

The postoperative development of cysts following osteochondral grafting procedures in the treatment of OCLs of the talus has been a matter of clinical concern.^38^ This concern arises from the hypothesis that cyst development could inherently lead to deterioration of the subchondral bone structure, vitality of the articular, transplanted or newly formed cartilage, and could subsequently lead to gradual deterioration of clinical outcomes. The findings of this study reveal that postoperative cysts are a commonly seen phenomenon after these procedures, but that the formation of cysts within the graft is not inherently linked to inferior clinical results. When comparing the literature of the ankle to that of the knee, The prevalence of postoperative cysts following surgical treatment of ankle OCLs is comparable in patients treated for knee OCLs, with rates ranging from 41% to 100% observed in the literature.^39-42^ Ackermann *et al*.^43^ described that the thickness of an osteochondral allograft is associated with subchondral cyst formation in the short-term, namely that thinner grafts potentially have a higher likelihood for the development of subchondral cysts. In a different study on OLTs which was excluded due to overlap in patients, no significant differences were found concerning the development of cysts between uncontained and contained lesions.^11^ In addition, Shimozono *et al*.^10^ proposed that potential cyst formation is due to the inferior host-graft integration. An interesting outcome, however, in the study by Shimozono *et al*.^10^ was that patients who received bone marrow aspirate concentrate (BMAC) after autologous osteochondral transplantation showed a significantly lower postoperative cyst occurrence rate than patients who did not receive BMAC (46% vs. 77%, with *P =* 0.022). In future research, it would therefore be of high interest to further explore the potential influence of biological adjuncts on the postoperative occurrence of cysts after osteochondral autograft, allograft, and osteoperiosteal transplantation.

The observed differences in cyst formation rates between autograft, allograft, and osteoperiosteal graft procedures were not statistically significant. This lack of significance suggests that the type of graft material utilized does not appear to be a major contributing factor to the development of postoperative cysts. In addition, allografts are possibly less vascularized, and this may contribute to more cyst formation if they are not fully incorporated in the talus. Future research with larger sample sizes could help elucidate whether there are any subtle differences that may not have been apparent in the present study.

It must be noted that cysts themselves are purely a radiological finding and might not be of clinical relevance. Therefore, it was the secondary aim of the present study to explore the correlation between the presence of postoperative cysts and clinical outcomes. The lack of a significant correlation between postoperative cysts and clinical outcomes could be interpreted in several ways. It is possible that the cysts that develop following these grafting procedures are largely asymptomatic and do not have a substantial impact on the function or subjective well-being of the patients. This may be due to the fact that the grafting procedures may be effective in restoring the biomechanical properties of the talus, even in the presence of cysts, resulting in favorable clinical outcomes. Another potential reason could be that the new graft is not fully innervated, and thus, it could not transmit pain signals. The clinical effect of cyst formation in the long-term may, however, be more of importance.

At longer-term follow-up, the biomechanical effects of the graft in the ankle may become more apparent. For example, it has been proposed that the subchondral bone is not communicating adequately with the articular joint space and that bone channels and thus cysts are formed.^44,45^ This may only become symptomatic at longer follow-ups. Possible sealing of the grafts with a fibrin sealant may be an option in that case. However, the clinical relevance is not known and should be investigated further before the routine recommendation of the use of adhesive sealant.

The management of postoperative cysts is another aspect to consider. The findings of this study suggest that the mere presence of these cysts may not warrant immediate intervention. However, it is essential to monitor patients for any potential symptoms or complications related to cysts, as some individuals may still experience discomfort or other issues. Identifying the characteristics of cysts that are more likely to become symptomatic and/or lead to failure of the graft together with developing guidelines for intervention based on these characteristics could be an important area for future research.

It is of importance to consider the potential limitations of the present study. First, the included studies were subject to variability in terms of sample size, follow-up duration, and the methods used to assess clinical outcomes. Furthermore, it should be noted that it is a limitation that it was not possible to perform a subanalysis on cyst occurrence in- and outside of the graft; this is due to the fact that solely Shimozono *et al*.^38^ reported on this outcome specifically. Such heterogeneity in the study design and assessment criteria may introduce biases or confounding factors that could influence the results. In addition, there is a possibility that the true clinical impact of postoperative cysts may be more subtle and require longer follow-up periods and larger sample sizes to become apparent. Moreover, studies using the MOCART score were excluded, as the question regarding cyst formation also includes edema. An important limitation that should also be mentioned here is the fact that it was not possible to assess the impact of body mass index (BMI), preoperative lesion size, size and number of grafts, potentially performed concomitant surgical procedures, as well as variations in postoperative rehabilitation protocols on outcomes due to the underreporting and heterogeneity in reporting of these data in the included studies.

The clinical implication of the present study is that cysts occur frequently in autograft, allograft, and osteoperiosteal graft procedures, but that these cysts alone are not of immediate concern. It must, however, be investigated further which patients develop cysts, and in which patients these cysts become symptomatic. This can help in the shared decision-making process by managing patients’ expectations.

Future research in this field could benefit from a more standardized approach to assessing clinical outcomes, radiological protocols as well as radiological scoring systems at follow-up, which would allow for better comparisons between different studies. As part of this, future research can also focus on a more in-depth analysis of the localization, morphology, and size of postoperative presence of cysts in order to shed more light on the etiology behind the occurrence of these cysts. Awareness of the high frequency may encourage future studies to include standardized assessment of cyst presence, location, and development over time. Moreover, longer-term follow-up studies could provide more insight into whether the presence of postoperative cysts has any delayed or cumulative effects on clinical outcomes, which may not be immediately evident.

## Conclusions

The occurrence of postoperative cysts is common following osteochondral autograft transplantation (42%), allograft transplantation (58%), and osteoperiosteal graft transplantation (34%) for OCLs of the talus—without significant intertreatment differences. However, the presence of postoperative cysts was not correlated with clinical outcomes in any of the studies. These findings suggest that postoperative cysts are common but may have limited clinical implications for patients and surgeons. Future research should assess whether the postoperative presence of cysts correlates with (clinical) outcomes at longer follow-up.

## Appendix

**Appendix A.** Search strategy. Search strategy until October 23, 2023.

PUBMED	Search Terms
#1	“Osteochondritis Dissecans”[Mesh]
#2	Osteochondritis dissecans[tiab] OR osteochondrosis dissecans[tiab] OR osteochondrolysis[tiab] OR OCD[tiab] OR OLT[tiab]
#3	(osteochondral[tiab] OR chondral[tiab] OR transchondral[tiab] OR cartilage*[tiab]) AND (defect*[tiab] OR lesion*[tiab])
#4	#1 OR #2 OR #3
#5	“Talus”[Mesh]
#6	talus[tiab] OR talar*[tiab] OR ankle[tiab]
#7	#5 OR #6
#8	#4 AND #7
EMBASE	Search terms
#1	(osteochondritis dissecans/or (osteochondritis dissecans or osteochondrosis dissecans or osteochondrolysis or OCD or OLT).ti,ab,kw. or ((osteochondral or chondral or osteochondral or transchondral or cartilage*) adj3 (defect* or lesion*)).ti,ab,kw.) and (talus/ or (talus or talar* or ankle).ti,ab,kw.)
COCHRANE	Search terms
#1	MeSH descriptor: [Osteochondritis Dissecans] explode all trees
#2	osteochondritis dissecans or osteochondrosis dissecans or osteochondrolysis or OCD or OLT:ti,ab,kw (Word variations have been searched)
#3	(osteochondral or chondral or transchondral or cartilage*) and (defect* or lesion*):ti,ab,kw (Word variations have been searched)
#4	#1 or #2 or #3
#5	MeSH descriptor: [Talus] explode all trees
#6	Talus or talar* or ankle*:ti,ab,kw (Word variations have been searched)
#7	#5 or #6

**Appendix B.** Outcomes of MINORS Assessment.

MINORS									Additional Criteria for Comparative Studies				
Study	A Clearly Stated Aim	Inclusion of Consecutive Patients	Prospective Collection of Data	Endpoint Appropiate to The Aim of The Study	Unbiased Assesment of The Study Endpoint	Follow-up Period Appropiate to The Aim of The Study	Lost of Follow-up Less Than 5%	Prospective Calculation of Study Size	An Adequate Control Group	Contemporary Group	Baseline Equivalent of Groups	Adequate Statistical Analysis	Total
Dahmen *et al*. (2023)	2	2	2	2	1	2	2	2	–	–	–	–	15/16
Fraser *et al*. (2016)	2	2	1	2	2	2	2	0	–	–	–	–	13/16
Haene *et al*. (2012)	1	0	2	1	2	2	2	0	–	–	–	–	10/16
Hansen *et al*. (2021)	2	2	1	2	1	2	2	0	2	2	2	2	20/24
Hu *et al*. (2013)	2	2	0	2	0	2	0	0	–	–	–	–	8/16
Imhoff *et al*. (2011)	2	2	2	2	1	1	1	0	–	–	–	–	11/16
Leumann *et al*. (2014)	1	2	2	1	1	2	1	0	–	–	–	–	10/16
Li *et al*. (2023)	1	2	2	2	1	2	1	0	–	–	–	–	11/16
(Ross *et al*. (2016)	2	0	1	2	2	1	2	0	–	–	–	–	9/16
Shimozono *et al*. (2018)	2	2	2	2	1	2	2	0	2	2	2	2	21/24
Valderrabano *et al*. (2009)	2	2	2	2	0	2	0	0	–	–	–	–	10/16
Wan *et al*. (2022)	2	2	1	2	2	1	2	0	–	–	–	–	12/16
Woelfle *et al*. (2013)	1	2	0	1	1	2	1	0	–	–	–	–	8/16
